# Is Whole‐Brain Radiotherapy for Brain Metastases an Overestimated Therapy? A Retrospective Study of Real‐World Data Using Landmark Analyses

**DOI:** 10.1002/cam4.70522

**Published:** 2024-12-20

**Authors:** Florian Hoelzl, Oliver Koelbl, Isabella Gruber

**Affiliations:** ^1^ University of Regensburg Regensburg Germany; ^2^ Department of Radiation Oncology University Hospital of Regensburg Regensburg Germany

**Keywords:** brain metastases, neurological symptoms, patient selection, whole‐brain radiotherapy

## Abstract

**Background:**

The role of whole‐brain radiotherapy for patients with brain metastases is changing as immunotherapy and molecularly targeted therapies advance. However, whole‐brain radiotherapy continues to be part of the multimodal concept.

**Methods:**

This retrospective study included 285 patients who received whole‐brain radiotherapy for brain metastases, using a median dose of 30 Gy. The study analyzed prognostic factors for survival using Cox regression analyses, while two landmark analyses, reflecting a minimum survival of 60 and 90 days, accounted for early deaths. Neurological symptoms were compared before and after treatment using the McNemar test.

**Results:**

The median patient age was 62 years. Non‐small cell lung cancer (*n* = 95), breast cancer (*n* = 53), and small cell lung cancer (*n* = 48) were the most frequent cancer types. Median survival was 4.3 months (interquartile range 1.8–11.1). In the multivariable Cox regression model, patients who received additional immunotherapy/molecularly targeted therapy had a higher chance of survival than others. Overall survival was influenced by control of primary cancer, extracranial metastases, age, Karnofsky performance status, and number of brain metastases. The 90‐day landmark analysis included 181 patients who survived at least 90 days, reflecting that 104 patients (36.5%) died within the first 90 days. The 90‐day landmark analysis confirmed all predictive variables for survival. Patients who died before the 90‐day landmark endpoint had more brain metastases, lower Karnofsky performance status, higher age, and were less frequently treated with immunotherapy/molecularly targeted therapy than those surviving at least 90 days. The treatment significantly improved neurological symptoms.

**Conclusion:**

These results indicate an insufficient patient selection, as one‐third of patients treated with whole‐brain radiotherapy died within 90 days. However, neurological symptoms improved, and the addition of immunotherapy and/or molecularly targeted therapy to whole‐brain radiotherapy was associated with better survival. Patients receiving whole‐brain irradiation should be more carefully selected.

**Trial Registration:**

ClinicalTrials: 24‐3626‐104

## 1 Introduction

Patients with malignant melanoma, and lung and breast cancer frequently develop brain metastases. While patients with favorable prognosis are typically referred directly for local therapies (e.g., surgery and/or stereotactic radiotherapy) or upfront immunotherapy/targeted therapies, patients with multiple brain metastases continue to receive whole‐brain radiotherapy [1, 2]. Currently, whole‐brain radiotherapy is increasingly delayed for patients with oligo‐symptomatic brain metastases and molecular targets amenable to systemic therapy [1, 2, 3, 4]. Drugs that penetrate the blood–brain barrier, targeting BRAF, EGFR, HER2, ALK, and ROS1, as well as immunotherapy (i.e., anti‐PD‐1 and anti‐CTLA4 checkpoint inhibitors), are increasingly applied, thus changing the role of whole‐brain radiotherapy [2, 4]. Additionally, stereotactic radiotherapy is preferred for patients with up to 10 brain metastases, withholding whole‐brain radiotherapy for those with extensive disease [1]. For patients with poor prognosis or performance status, the best supportive care is recommended. However, whole‐brain radiotherapy is included in this concept to improve neurological symptoms [5]. Yet, guidelines recommend a life expectancy of about 3 months to perform whole‐brain radiotherapy instead of the best supportive care [2].

The response of brain metastases to whole‐brain radiotherapy takes several weeks, but some patients may die prematurely from extracranial metastases or their primary disease. Therefore, selecting patients who are likely to benefit from whole‐brain irradiation, beyond symptom relief, is of significant interest. This retrospective study analyzed the survival of patients with brain metastases from solid tumors and identified prognostic factors for survival using Cox regression and landmark analyses at specific time points (minimum survival of 60 and 90 days). Although the expected overall survival is limited, symptom relief is relevant. Thus, the present study also analyzed and compared neurological symptoms in patients before and immediately after whole‐brain radiotherapy.

## 2 Methods and Materials

### 2.1 Patients

A total of 319 patients received whole‐brain radiotherapy for brain metastases from solid tumors at the Department of Radiation Oncology of the University Hospital of Regensburg between June 2013 and December 2023. Twenty‐one patients did not complete whole‐brain radiotherapy before reaching the minimum dose of 27 Gy. Additionally, patients were excluded due to patient age < 18 years (one patient), moving abroad (one patient), pretherapeutic computed tomography scans without contrast medium (three patients), missing medical records (two patients), and due to whole‐brain radiotherapy administered with adjuvant intent (*n* = 6), resulting in a final cohort of 285 participants. The study documented diagnoses and divided them into radio‐resistant cancers (malignant melanoma, renal cell cancer, and sarcoma) and other cancer types. The study captured patient age, sex, Karnofsky performance status, control of primary cancer, presence of extracranial metastases, and the number of brain metastases at the time of whole‐brain radiotherapy. Control of the primary cancer was defined as a primary cancer that had either been surgically resected or exhibited stable disease (including locoregional lymph nodes) following radiotherapy, as confirmed at the most recent staging. Prognostic scores were determined on the day of whole‐brain radiotherapy, as described in the original scoring systems [6, 7]. The use of cytotoxic chemotherapy, immunotherapy, or molecularly targeted therapy was determined by the oncologists and was recorded as performed either during whole‐brain radiotherapy or within 6 weeks before or after initiation of whole‐brain radiotherapy. Data on whole‐brain radiotherapy (including single dose, total dose, boost irradiation, and treatment duration) and intervals between specific dates were extracted from the medical records of the University Hospital of Regensburg. The study analyzed the neurological symptom burden of patients receiving whole‐brain radiotherapy. Symptomatic patients were defined as those presenting with symptoms related to brain metastases, regardless of systemic steroid medication use. Patients were categorized as asymptomatic or symptomatic on the first and last day of whole‐brain radiotherapy. Symptoms recorded included headaches, emesis, focal neurological deficits, cognitive disorders (memory, concentration, attention, thinking problems), epileptic seizures, and dizziness. Data collection concluded in September 2024. The local Ethics Board of the University of Regensburg approved this retrospective study (approval number 24‐3626‐104, dated 26 January 2024).

### 2.2 Whole‐Brain Radiotherapy

The median time from diagnosis of brain metastases to initiation of whole‐brain radiotherapy was 18 days (IQR 8.5–43.0 days, mean 80.0 days, SD 179.7 days), while the median duration of whole‐brain radiotherapy was 16 days (IQR 13.0–19.0 days, mean 16.7 days, SD 3.9 days). More than half of the patients (*n* = 160, 56.1%) were hospitalized during treatment, while 43.9% (*n* = 125) of patients received whole‐brain radiotherapy on an outpatient basis. Surgical resection of at least one brain metastasis was performed in 69 patients (24.2%) prior to the initiation of whole‐brain radiotherapy. The frequencies of brain metastasis resections (*n* = 69) performed prior to whole‐brain radiotherapy did not differ among the various primary diagnoses (*p* = 0.095). With respect to the individual primary diagnoses, resections were performed in CUP (*n* = 4, 4/10, 40.0%), gastrointestinal cancer (*n* = 4, 4/11, 36.4%), NSCLC adenocarcinoma (*n* = 25, 25/75, 33.3%), NSCLC nonadenocarcinoma (*n* = 5, 5/20, 25.0%), malignant melanoma (*n* = 11, 11/45, 24.4%), breast cancer (*n* = 9, 9/53, 17.0%), SCLC (*n* = 5, 5/48, 10.4%), and other cancer types (*n* = 6, 6/23, 26.1%). Similarly, when divided into nonradioresistant (*n* = 233) and radioresistant cancer types (malignant melanoma, sarcoma, and renal cell cancer; *n* = 52), resections of at least one brain metastasis were performed at comparable frequencies prior to whole‐brain radiotherapy (radioresistant, *n* = 14, 14/52, 26.9% vs. nonradioresistant, *n* = 55, 55/233, 23.6%; *p* = 0.596).

A total of 246 patients (86.3%) received whole‐brain radiotherapy without a history of prior local radiotherapy, while 39 patients (13.7%) had undergone local radiotherapy for brain metastases before whole‐brain radiotherapy. Most patients (*n* = 246, 86.3%) were treated with systemic dexamethasone during, or within 6 weeks before or after whole‐brain radiotherapy.

Whole‐brain radiotherapy was delivered using 3D techniques with 15 MV photons generated by linear accelerators, while boost radiotherapy was performed in intensity‐modulated radiotherapy (IMRT) and volumetric‐modulated arc therapy (VMAT) techniques. Memantine and hippocampal dose reduction were not standard practices. Patients received whole‐brain radiotherapy with a total median dose of 30.0 Gy (IQR 30.0–35.0 Gy), using single doses of 3.0 Gy (IQR 2.5–3.0 Gy). More than half of the patients (*n* = 188, 66.0%) received whole‐brain radiotherapy without boost radiotherapy, while 97 patients (34.0%) received a simultaneous or sequential boost targeting single brain metastases. Of these, 39 patients were treated with simultaneous integrated boost, receiving a median single dose of 3.5 Gy (IQR 3.0–3.9 Gy) to a total median dose of 42.0 Gy (IQR 39.0–43.0 Gy). Fifty‐eight patients received a sequential boost, with a median single dose of 3.0 Gy (IQR 3.0–3.0 Gy) to a median total boost dose of 9.0 Gy (IQR 9.0–15.0 Gy), resulting in a median total dose of 44 Gy (IQR 39.0–45.0 Gy).

Surgical resection of brain metastases was performed in seven patients (2.5%) after whole‐brain radiotherapy. Additionally, 16 patients (5.6%) received local radiotherapy for brain metastases following whole‐brain radiotherapy.

### 2.3 Systemic Pharmacotherapies

The majority of patients (*n* = 221, 77.5%) received cytotoxic chemotherapy, immune checkpoint inhibitors, antihormonal therapies, or molecularly targeted therapies either during whole‐brain radiotherapy or within 6 weeks before or after the initiation of whole‐brain radiotherapy. In contrast, 64 patients (22.5%) did not receive any systemic pharmacotherapy during the specified period. Among those who received systemic pharmacotherapy, 146 patients were administered molecularly targeted therapies and/or immune checkpoint inhibitors, either alone or in combination with other therapies. Detailed information on the systemic therapies is presented in Table 1.

**TABLE 1:** Systemic pharmacotherapies administered during whole‐brain radiotherapy or within 6 weeks before or after the initiation of treatment.

	*n* (%)
No systemic therapy	64 (22.5)
Cytotoxic chemotherapy alone	69 (24.2)
Molecularly targeted therapies alone	37 (13.0)
Immune checkpoint inhibitors + cytotoxic chemotherapy	35 (12.3)
Immune checkpoint inhibitors alone	33 (11.6)
Cytotoxic chemotherapy + molecularly targeted therapies	25 (8.8)
Antihormonal therapy alone	6 (2.1)
Immune checkpoint inhibitors + molecularly targeted therapies	6 (2.1)
Other combination with immune checkpoint inhibitors or molecularly targeted therapies	10 (3.5)

The immune checkpoint inhibitors included atezolizumab, nivolumab, ipilimumab, pembrolizumab, and durvalumab. The molecularly targeted therapies consisted of bevacizumab, trastuzumab, pertuzumab, abemaciclib, alpelisib, capmatinib, palbociclib, herceptin, neratinib, T‐DM1, dabrafenib, trametinib, binimetinib, encorafenib, cobimetinib, gefitinib, afatinib, erlotinib, nintedanib, osimertinib, amivantamab, temsirolimus, sorafenib, cabozantinib, and sunitinib.

### 2.4 Statistical Methods

Patient characteristics are presented as median, interquartile range (IQR), mean, and standard deviation (SD) for continuous variables and as absolute and relative frequencies for categorical variables. The Mann–Whitney *U*‐test was used for comparisons of continuous variables, and the chi‐square test of independence for categorical variables. The McNemar test examined changes in the frequencies of neurological symptoms before and after treatment. Overall survival was calculated from the first day of whole‐brain radiotherapy to the date of death or last follow‐up using the Kaplan–Meier method. The log‐rank test compared overall survival between groups. Overall survival was analyzed by uni‐ and multivariable Cox proportional hazard regression models. Hazard ratio (HR) and 95% confidence interval (95% CI) are presented as effect estimates. The proportional hazard assumption of the Cox models was tested by using Schoenfeld‐type residuals. Two landmark analyses were performed as some patients died immediately after completion of therapy and before initiation of sequential systemic therapy. The landmark analysis included patients with a minimum survival of 60 and 90 days after initiation of whole‐brain radiotherapy reflecting a survival time of at least 60 and 90 days after initiation of whole‐brain radiotherapy. A two‐sided *p*‐value < 0.05 was considered statistically significant. Statistical analysis was performed using SPSS 26.0 (SPSS Inc., Chicago, IL, USA) and R, version 4.1.2.

## 3 Results

### 3.1 Patient Characteristics

The median follow‐up from the date of diagnosis of brain metastases to the last follow‐up or death was 7.1 months (IQR 2.7–14.3 months, mean 12.4 months, SD 16.2 months). Patients developed brain metastases at a median of 4.7 months (IQR, −0.03 to 26.4 months, mean 22.4 months, SD 40.1 months) after their initial cancer diagnosis, indicating that, in some cases, brain metastases were diagnosed before histological confirmation of the primary tumor. Table 2 summarizes patient and disease characteristics.

**TABLE 2:** Patient characteristics (*n* = 285).

	Value
Histology, *n* (%)	
Non‐small cell lung cancer (NSCLC), adenocarcinoma	75 (26.3)
Non‐small cell lung cancer (NSCLC), nonadenocarcinoma	20 (7.0)
Breast cancer	53 (18.6)
Small cell lung cancer (SCLC)	48 (16.8)
Malignant melanoma	45 (15.8)
Gastrointestinal cancer	11 (3.9)
Cancer of unknown primary (CUP)	10 (3.5)
Other	23 (8.1)
Patient age at the time of whole‐brain radiotherapy (years)	
Median (interquartile range, IQR)	61.8 (IQR 55.9–70.7)
Mean (standard deviation, SD)	62.3 (SD 11.3)
Sex, *n* (%)	
Female	144 (50.5)
Male	141 (49.5)
Status of the primary cancer^a^, *n* (%)	
No control	127 (44.6)
Control	158 (55.4)
Extracerebral metastases, *n* (%)	
Present	240 (84.2)
Absent	45 (15.8)
Karnofsky performance status	
Median (interquartile range, IQR)	70 (IQR 50–80)
Mean (standard deviation, SD)	65.4 (SD 15.4)
Karnofsky performance status (%)	
90–100	36 (12.6)
70–80	109 (38.2)
< 70	140 (49.1)
Graded prognostic assessment, GPA at the time of whole‐brain radiotherapy	
Median (interquartile range, IQR)	1.0 (IQR 0.5–1.5)
Mean (standard deviation, SD)	0.9 (SD 0.7)
Diagnosis‐specific graded prognostic assessment, dsGPA at the time of whole‐brain radiotherapy	
Median (interquartile range, IQR)	1.0 (IQR 0.5–2.0)
Mean (standard deviation, SD)	1.2 (SD 0.8)
Recursive partitioning analysis, RPA at the time of whole‐brain radiotherapy	
Median (interquartile range, IQR)	3.0 (IQR 2.0–3.0)
Mean (standard deviation, SD)	2.5 (SD 0.6)
Localization of brain metastases, *n* (%)	
Supratentorial and infratentorial	216 (75.8)
Supratentorial	62 (21.8)
Infratentorial	7 (2.5)
Number of brain metastases, *n* (%)	
1–5 brain metastases	89 (31.2)
> 5 brain metastases and/or leptomeningeal spread	196 (68.8)
Molecularly targeted therapy or immunotherapy, during, or within 6 weeks before or after the initiation of whole‐brain radiotherapy, *n* (%)	
Yes	146 (51.2)
No	139 (48.8)

^a^ Control of primary cancer, a controlled primary cancer was defined as one that had either been surgically resected or exhibited stable disease (including locoregional lymph nodes) following radiotherapy, as confirmed at the most recent staging.

Patients received whole‐brain radiotherapy for non‐small cell lung cancer (NSCLC; *n* = 95, 33.3%), breast cancer (*n* = 53, 18.6%), small cell lung cancer (SCLC; *n* = 48, 16.8%), malignant melanoma (*n* = 45, 15.8%), and gastrointestinal cancer (*n* = 11, 3.9%). Malignant melanoma, renal cell cancer (*n* = 5), and sarcoma (*n* = 2) were classified as radio‐resistant cancer types. Extracranial metastases were present in 240 patients (84.2%), while 45 (15.8%) had brain metastases only. The median Karnofsky performance status at the time of whole‐brain radiotherapy was 70 (IQR 50–80). Additionally, half of the patients (*n* = 146, 51.2%) received immunotherapy and/or molecularly targeted therapy during, or within 6 weeks before or after the initiation of whole‐brain radiotherapy (Table 2).

The use of immunotherapy and/or molecularly targeted therapy during, or within 6 weeks before or after the initiation of whole‐brain radiotherapy varied across the primary diagnoses (*p* < 0.001): malignant melanoma (*n* = 36, 80.0%), NSCLC adenocarcinoma (*n* = 41, 54.7%), NSCLC nonadenocarcinoma (*n* = 10, 50.0%), SCLC (*n* = 21, 43.8%), breast cancer (*n* = 29, 54.7%), and other cancer types (*n* = 9, 20.5%). Four patients with CUP received platinum‐based chemotherapy during or within 6 weeks before or after whole‐brain radiotherapy, without immuno‐ or molecularly targeted therapies. The median patient age was not different between patients receiving immunotherapy or molecularly targeted therapy during, or within 6 weeks before or after whole‐brain radiotherapy (60.8 years, IQR 54.4–70.9 years, mean 62.0 years, SD 11.4 years) and those who did not (63.4 years, IQR 57.3–70.6 years, mean 62.9 years, SD 11.2 years; *p* = 0.282). The median Karnofsky performance status was 60 (IQR 50–70) in patients who did not receive immunotherapy and/or molecularly targeted therapy, compared to 70 (IQR 60–80) in those who received these therapies (*p* < 0.001).

### 3.2 Overall Survival

**FIGURE 1:**
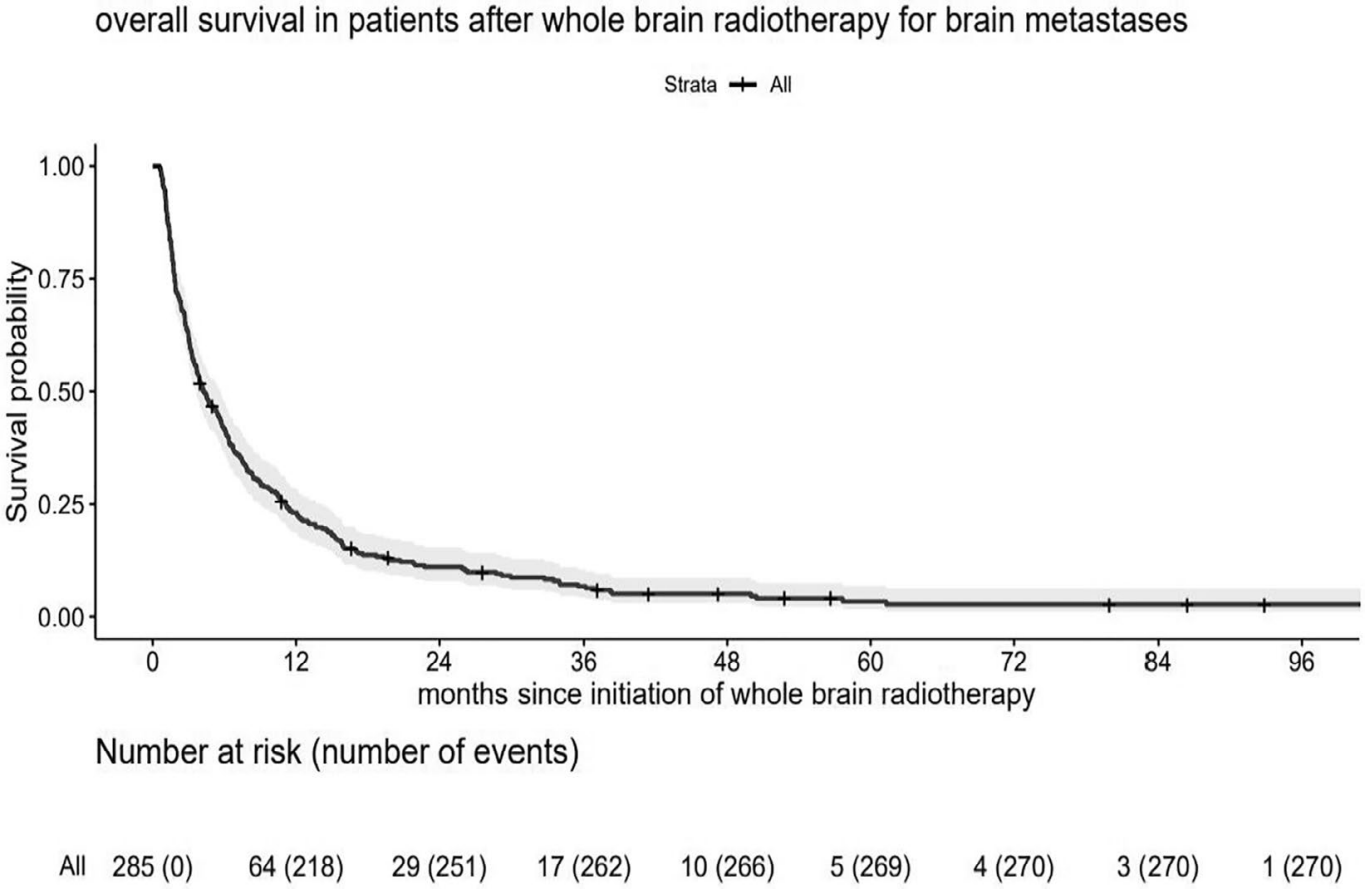
Overall survival in patients after whole‐brain radiotherapy (*n* = 285).

The median survival of all patients was 4.3 months (IQR 1.8–11.1 months) after initiation of whole‐brain radiotherapy (Figure 1). Kaplan–Meier estimates of 1‐ and 2‐year survival was 23.1% and 11.0%, respectively.

The median overall survival was 6.4 months (IQR 2.3–9.0 months) in cancer of unknown primary (CUP), 5.6 months (IQR 2.4–14.2 months) in breast cancer, 4.4 months (IQR 1.7–10.0 months) in malignant melanoma, 4.7 months (IQR 2.4–8.7 months) in small cell lung cancer (SCLC), 4.1 months (IQR 1.6–11.7 months) in non‐small cell lung cancer (NSCLC), 2.2 months (IQR 1.7–3.2 months) in gastrointestinal cancers, and 3.3 months (IQR 1.8–12.2 months) in other cancer types. There were no significant differences in median survival times between all cancer types (log‐rank, *p* = 0.614).

The median survival was 6.4 months (IQR 2.8–16.9 months) for patients who received immunotherapy and/or molecularly targeted therapy (*n* = 146) during, or within 6 weeks before or after the initiation of whole‐brain radiotherapy, compared to 3.1 months (IQR 1.4–6.8 months) for those who did not receive these therapies during the specified period (*n* = 139). This difference was statistically significant (*p* < 0.001). Estimated 1‐ and 2‐year survival rates were 34.3% and 18.3% for patients receiving immunotherapy and/or molecularly targeted therapy during the specified period, compared to 11.5% and 3.6% for those who did not receive these therapies.

### 3.3 Univariable and Multivariable Regression Analyses

Table 3 summarizes the results of the univariable and multivariable analyses of overall survival. Patients without control of primary cancer had a lower chance of overall survival (HR 1.314, 95% CI 1.026–1.682, *p* = 0.031) compared to those with controlled primary cancer. Additional factors negatively impacting overall survival included the presence of extracranial metastases (HR 1.830, 95% CI 1.267–2.644, *p* = 0.001), patient age > 65 years (HR 1.614, 95% CI 1.249–2.087, *p* < 0.001), and a Karnofsky performance status of < 70 (HR 2.262, 95% CI 1.734–2.950, *p* < 0.001). Patients who received molecularly targeted therapy and/or immunotherapy during, or within 6 weeks before or after the initiation of whole‐brain radiotherapy had a higher chance of overall survival compared to those not receiving molecularly targeted therapy and/or immunotherapy (HR 0.465, 95% CI 0.356–0.606, *p* < 0.001). Patients presenting with > 5 brain metastases had a lower chance of survival (HR 1.399, 95% CI 1.054–1.856, *p* = 0.020) compared to those presenting with 1–5 brain metastases at the time of whole‐brain radiotherapy. Histology showed no association with overall survival.

**TABLE 3:** Univariable and multivariable Cox regression analysis of overall survival after whole‐brain radiotherapy for brain metastases (*n* = 285).

	Univariable model			Multivariable model		
	HR	95% CI	*p*	HR	95% CI	*p*
Radioresistant cancer types: Malignant melanoma, renal cell cancer, sarcoma	0.946	0.740–1.382	0.946	1.362	0.976–1.901	0.070
No control of primary cancer^a^	1.057	1.057–1.711	0.016*	1.314	1.026–1.682	0.031*
Presence of extracranial metastases	1.835	1.286–2.618	< 0.001***	1.830	1.267–2.644	0.001**
Patient age						
≤ 65 years (reference)						
> 65 years	1.592	1.245–2.036	< 0.001***	1.614	1.249–2.087	< 0.001***
Number of brain metastases						
1–5 (reference)						
> 5 and/or leptomeningeal spread	1.465	1.126–1.906	0.004**	1.399	1.054–1.856	0.020*
Karnofsky performance status						
70–100 (reference)						
< 70	2.724	2.116–3.505	< 0.001****	2.262	1.734–2.950	< 0.001****
Molecularly targeted and/or immunotherapy during, or within 6 weeks before or after the initiation of whole‐brain radiotherapy	0.497	0.388–0.635	< 0.001****	0.465	0.356–0.606	< 0.001****

^a^ Control of primary cancer, a controlled primary cancer was defined as one that had either been surgically resected or exhibited stable disease (including locoregional lymph nodes) following radiotherapy, as confirmed at the most recent staging. *p < 0.05, **p < 0.01, ***p < 0.001, ****p < 0.0001.

### 3.4 Landmark Analysis at the 60‐Day Landmark Endpoint

Some patients died immediately after whole‐brain radiotherapy. Therefore, we performed a landmark analysis that included patients with a minimum survival of 60 days (*n* = 206). The median survival was 7.1 months (IQR 3.7–15.0 months). The results of the multivariable Cox regression analysis were similar to the results of Table 3.

### 3.5 Landmark Analysis at the 90‐Day Landmark Endpoint

A second landmark analysis was conducted, focusing on patients who survived a minimum of 90 days, equivalent to at least 3 months of survival following the initiation of whole‐brain radiotherapy. The 90‐day landmark analysis included 181 patients (63.5%) who survived at least 90 days, while 104 patients (36.5%) who died within the first 90 days were excluded. Among patients who met the 90‐day survival threshold (*n* = 181), the median survival was 8.3 months (IQR 4.7–15.9 months). Comparing the characteristics of patients included in the 90‐day landmark analysis with those excluded (prematurely deceased) revealed several differences. Patients who died before reaching the 90‐day endpoint were older (median 65.0 years, IQR 57.7–73.8 years, mean 65.3 years, SD 10.4 years) than those who survived at least 90 days (median 60.7 years, IQR 54.7–69.1 years, mean 60.8 years, SD 11.5 years; *p* = 0.003). The 90‐day survivors also had a better Karnofsky performance status (median 70, IQR 60–80) than those who died prematurely (median 60, IQR 50–60, *p* < 0.001). Dexamethasone was more frequently administered to excluded patients than to included patients (excluded patients: *n* = 98, 94.2% vs. included patients: *n* = 148, 81.8%; *p* = 0.004). Although extracranial metastases were similarly distributed between groups, there was a numerical trend suggesting a higher prevalence among excluded patients (excluded patients: *n* = 93, 89.4%; included patients: *n* = 147, 81.2%; *p* = 0.091). Patients who died prematurely had more brain metastases (> 5 brain metastases) than those who survived at least 90 days (*n* = 80, 76.9%, vs. *n* = 116, 64.1%; *p* = 0.025). Immunotherapy and/or molecularly targeted therapy were more commonly administered in patients with a minimum survival of 90 days compared to excluded patients (included patients: *n* = 109, 60.2% vs. excluded patients: *n* = 37, 35.6%; *p* < 0.001). Patients included in the landmark analysis underwent resection of brain metastases prior to whole‐brain radiotherapy just as frequently as the excluded patients (included patients: *n* = 44, 24.3% vs. excluded patients: *n* = 25, 24.0%; *p* = 1.00). The distribution of cancer diagnoses, including radio‐resistant types, and the control of the primary cancer did not differ significantly between groups. The multivariable Cox regression model for the 90‐day minimum survival group, shown in Table 4, yielded results consistent with the overall findings presented in Table 3.

**TABLE 4:** Ninety‐day landmark analysis: Multivariable Cox regression analysis of overall survival after whole‐brain radiotherapy for brain metastases (*n* = 181).

Ninety‐day landmark analysis	Multivariable model		
	HR	95% CI	*p*
Radioresistant cancer types: Malignant melanoma, renal cell cancer, sarcoma	1.197	0.774–1.852	0.418
No control of primary cancer^a^	1.526	1.107–2.103	0.010**
Presence of extracranial metastases	1.675	1.066–2.632	0.025*
Patient age			
≤ 65 years (reference)			
> 65 years	1.838	1.317–2.567	< 0.001***
Number of brain metastases			
1–5 (reference)			
> 5 and/or leptomeningeal spread	1.496	1.049–2.134	0.026*
Karnofsky performance status			
70–100 (reference)			
< 70	1.703	1.202–2.412	0.003**
Molecularly targeted and/or immunotherapy during, or within 6 weeks before or after the initiation of whole‐brain radiotherapy	0.495	0.351–0.699	< 0.001****

^a^ Control of primary cancer, a controlled primary cancer was defined as one that had either been surgically resected or exhibited stable disease (including locoregional lymph nodes) following radiotherapy, as confirmed at the most recent staging. *p < 0.05, **p < 0.01, ***p < 0.001, ****p < 0.0001.

**FIGURE 2:**
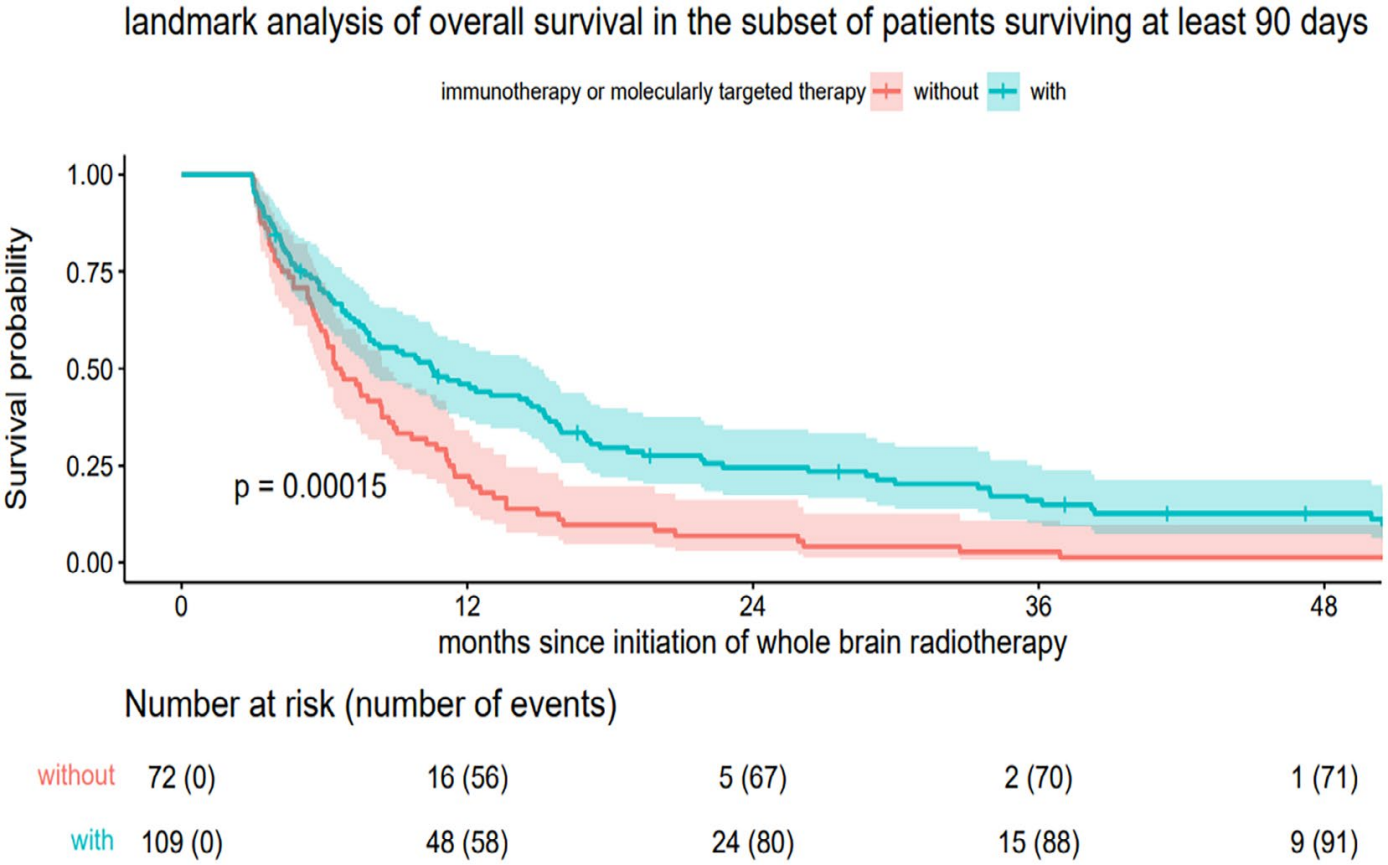
Landmark analysis of overall survival in patients with a minimum survival of 90 days, stratified by the administration of immunotherapy and/or molecularly targeted therapy during, or within 6 weeks before or after the initiation of whole‐brain radiotherapy (*n* = 181).

Figure 2 illustrates the survival curves for patients who survived at least 90 days (90‐day landmark analysis), stratified by the administration of systemic therapy (immunotherapy and/or molecularly targeted therapy) during, or within 6 weeks before or after the initiation of whole‐brain radiotherapy (*n* = 181). The median survival time was 10.5 months (IQR 5.2–22.7 months) for patients who received immunotherapy and/or molecularly targeted therapy (*n* = 109), compared to 6.5 months (IQR 4.2–11.2 months) for those who did not receive these therapies (*n* = 72, *p* < 0.001). Kaplan–Meier estimates of 1‐ and 2‐year survival were 46.0% and 24.5%, respectively, for patients receiving immunotherapy and/or molecularly targeted therapy. In contrast, patients who did not receive immunotherapy and/or molecularly targeted therapy had a 1‐ and 2‐year survival of 22.2% and 6.9%, respectively. At the time of the last follow‐up, all surviving patients (*n* = 14) were receiving ongoing immunotherapy and/or molecularly targeted therapy.

### 3.6 Neurological Symptoms

Before the initiation of whole‐brain radiotherapy, 227 patients (79.6%) were symptomatic (at least one neurological symptom), while 58 patients (20.4%) were symptom‐free. Patients presented with a median of two neurological symptoms (IQR 1.0–3.0) prior to whole‐brain radiotherapy. Patients with at least one neurological symptom had more frequent therapy with dexamethasone (*n* = 207) compared to symptom‐free patients (*n* = 39, *p* < 0.001). Patients suffered from focal neurological deficits (*n* = 139, 48.8%), cognitive disorders (*n* = 86, 30.2%), dizziness/vertigo (*n* = 89, 31.2%), headache (*n* = 78, 27.4%), or nausea (*n* = 59, 20.7%). Epileptic seizures were present in 42 patients (14.7%) within 1 month prior to the start of whole‐brain radiotherapy (Table 5). Patients with nausea (*n* = 57, 96.9% vs. *n* = 2, 3.4%; *p* = 0.009), focal neurological deficits (*n* = 134, 96.4% vs. *n* = 5, 3.6%; *p* < 0.001), cognitive disorders (*n* = 81, 94.2% vs. *n* = 5, 5.8%; *p* = 0.014), and dizziness/vertigo (*n* = 84, 94.4% vs. *n* = 5, 5.6%; *p* = 0.008) had more frequent a therapy with dexamethasone compared to those without these symptoms.

**TABLE 5:** Neurological symptoms before and after whole‐brain radiotherapy (*n* = 285).

Symptoms	Before whole‐brain radiotherapy		On the last day of whole‐brain radiotherapy		*p* (McNemar)
	*n*	%	*n*	%	
Headaches					
Absent	207	72.6	263	92.3	< 0.001
Present	78	27.4	22	7.7	
Nausea					
Absent	226	79.3	264	92.6	< 0.001
Present	59	20.7	21	7.4	
Focal neurological deficit					
Absent	146	51.2	192	67.4	< 0.001
Present	139	48.8	93	32.6	
Cognitive disorders^a^					
Absent	199	69.8	231	81.1	< 0.001
Present	86	30.2	54	18.9	
Epileptic seizures^b^					
Absent	243	85.3	280	98.2	< 0.001
Present	42	14.7	5	1.8	
Dizziness/vertigo					
Absent	196	68.8	244	85.6	< 0.001
Present	89	31.2	41	14.4	

^a^ Cognitive disorders comprised problems in memory, concentration, attention, and thinking.

^b^ Epileptic seizures before whole‐brain radiotherapy were captured within 1 month before whole‐brain radiotherapy.

On the last day of whole‐brain radiotherapy, 139 patients (48.8%) were symptom‐free, while 146 (51.2%) patients had at least one neurological symptom. Table 5 shows the results of the McNemar test. Therapy had a significant effect, improving headaches (pre, *n* = 78; post, *n* = 22), nausea (pre, *n* = 59; post, *n* = 21), focal neurological deficits (pre, *n* = 139; post, *n* = 93), cognitive disorders (pre, *n* = 86; post, *n* = 54), epileptic seizures (pre, *n* = 42; post, *n* = 5), and dizziness/vertigo (pre, *n* = 89; post, *n* = 41).

## 4 Discussion

Literature indicates that the median survival for patients with multiple brain metastases is 3–6 months after whole‐brain radiotherapy, with patient characteristics and systemic therapy influencing the outcomes [4, 8]. The present study revealed a median survival of 4.3 months (IQR 1.8–11.1 months) for the overall patient population, with 1‐ and 2‐year survival rates of 23.1% and 11.0%, respectively. There were no significant differences in median survival times between all cancer types. We hypothesize that the absence of a detectable primary tumor may have contributed to the observed survival in patients with CUP (median 6.4 months). Unlike patients with NSCLC, who may die from complications related to the primary tumor, patients with CUP do not have a local tumor that could contribute to complications or early mortality. However, the small sample size of CUP patients (*n* = 10) introduces a bias that may influence these findings.

The multivariable regression model in the present study confirmed several risk factors associated with a decreased chance of survival after whole‐brain radiotherapy. Patients with extracranial metastases, higher patient age (> 65 years), higher number of brain metastases (> 5 brain metastases) or a lower Karnofsky performance status (< 70) had a reduced chance for survival. Conversely, a controlled primary cancer at the time of whole‐brain radiotherapy translated into a higher chance of survival. Notably, patients who received molecularly targeted therapy and/or immunotherapy during, or within 6 weeks before or after the initiation of whole‐brain radiotherapy had longer survival compared to those who did not receive these treatments. This benefit in survival was confirmed in the 90‐day landmark analysis, which excluded patients with premature deaths. Patients who received immunotherapy and/or molecularly targeted therapy during, or within 6 weeks before or after the initiation of whole‐brain radiotherapy and survived at least 90 days had a median survival time of 10.5 months (IQR 5.2–22.7 months). This underscores the importance of systemic therapy in controlling disease [4]. International guidelines recommend whole‐brain irradiation instead of the best supportive care for patients with a life expectancy of at least 3 months [2]. However, 36.5% (*n* = 104) of patients treated with whole‐brain radiotherapy died before the 90‐day landmark, indicating suboptimal pretherapeutic patient selection. Data from the 90‐day landmark analysis highlighted that older patients, those with poor Karnofsky performance status, and those with more than five brain metastases were prone to die before reaching the 90‐day milestone. Studies focusing on quality of life and symptom burden after whole‐brain radiotherapy for brain metastases indicate significant reductions in headaches, nausea, visual disorders, seizures, and motor dysfunction [9]. However, fatigue and lethargy may increase [9]. The present study found an improvement in all neurological symptoms by the last day of whole‐brain radiotherapy. Since most patients received a combination of whole‐brain irradiation, systemic therapy, and, to a large extent, dexamethasone, it is difficult to attribute symptom relief solely to radiotherapy. However, we identified at least one Radiation Therapy Oncology Group (RTOG) study suggesting that the symptom improvement after whole‐brain radiotherapy was similar for patients who received steroid medications and those who did not [10]. The Quality of Life after Treatment for Brain Metastases (QUARTZ) study [5] analyzed NSCLC patients with poor prognosis and brain metastases who were not suitable for resection or stereotactic radiotherapy. Patients were randomized to receive either whole‐brain radiotherapy plus dexamethasone (20 Gy in 5 fractions) or dexamethasone alone, without simultaneous systemic treatment. Median survival was similar between groups, with 9 weeks for patients receiving whole‐brain radiotherapy plus dexamethasone and 8.5 weeks for those omitting whole‐brain radiotherapy [5], highlighting the importance of combining whole‐brain radiotherapy with CNS‐active therapy. Literature indicates that whole‐brain radiotherapy has a limited impact on local control of brain metastases, especially in comparison to stereotactic radiotherapy. However, as demonstrated in the present study, whole‐brain radiotherapy significantly reduces neurological symptom burden.

This study is primarily limited by its retrospective design. The study categorized the number of brain metastases as 1–5 versus > 5, as the exact count was often unmeasurable in cases with a miliary spread of metastases. Additionally, the study did not analyze the volume of the brain metastases or assess the response of individual brain metastases to whole‐brain radiotherapy. As a result, progression‐free survival data could not be provided. The study did not investigate the causes of death, as determining the exact cause in patients with multiple metastases and end‐stage tumor diseases is challenging unless autopsy reports are available. The study did not capture patient‐reported quality of life or neurocognitive function after whole‐brain radiotherapy and whole‐brain radiotherapy with hippocampus avoidance was not standard, so no conclusions can be made regarding neurocognitive toxicity. Finally, the study lacks a comparison arm of patients receiving the best supportive care, preventing a comprehensive evaluation of which patients truly benefit from whole‐brain radiotherapy.

## 5 Conclusions

The present study revealed that one‐third of patients treated with whole‐brain radiotherapy died within 90 days of treatment, highlighting ongoing challenges in pretherapeutic selection of patients who are likely to benefit from the therapy. However, patients who received whole‐brain radiotherapy in combination with immunotherapy and/or molecularly targeted therapy during, or within 6 weeks before or after whole‐brain radiotherapy had better survival outcomes compared to those treated with whole‐brain radiotherapy alone. The data, however, cannot clarify whether the survival benefit was due to a reduction in extracranial tumor burden or a direct effect on intracranial metastases. We conclude that patients receiving whole‐brain irradiation should be more carefully selected, and molecular markers of radiation resistance may aid in this process.

## Author Contributions

**Florian Hoelzl:** conceptualization (equal), formal analysis (equal), writing – review and editing (equal). **Oliver Koelbl:** resources (equal), conceptualization (equal), methodology (equal), supervision (equal), writing – review and editing (equal). **Isabella Gruber:** data curation (equal), conceptualization (equal), formal analysis (equal), visualization (equal), methodology (equal), supervision (equal), validation (equal), writing – original draft (equal), writing – review and editing (equal).

## Ethics Statement

The local Ethics Board of the University of Regensburg approved this study (approval number, 24‐3626‐104, date, 26 January 2024). The study was conducted according to the guidelines of the Declaration of Helsinki.

## Consent

All patients gave written informed consent.

## Conflicts of Interest

The authors declare no conflicts of interest.

## Data Availability

The data that support the findings of this study are available on request from the corresponding author. The data are not publicly available due to privacy or ethical restrictions.
